# Assessment of LV ejection fraction using real-time 3D echocardiography in daily practice: direct comparison of the volumetric and speckle tracking methodologies to CMR

**DOI:** 10.1007/s12471-014-0577-1

**Published:** 2014-08-21

**Authors:** M. M. P. Driessen, E. Kort, M. J. M. Cramer, P. A. Doevendans, M. J. Angevaare, T. Leiner, F. J. Meijboom, S. A. J. Chamuleau, G. Tj Sieswerda

**Affiliations:** 1 Department of Cardiology, University Medical Centre Utrecht, Heidelberglaan 100, 3584 CX Utrecht, the Netherlands; 2 Interuniversity Cardiology Institute of the Netherlands (ICIN), Netherlands Heart Institute, Catharijnesingel 52, 3511 GC Utrecht, the Netherlands; 3 Department of Radiology, University Medical Centre Utrecht, Heidelberglaan 100, 3584 CX Utrecht, the Netherlands

**Keywords:** Real-time 3D echocardiography, Daily practice, Observer experience, CMR

## Abstract

**Aims:**

This study is the first to directly compare two widely used real-time 3D echocardiography (RT3DE) methods of cardiac magnetic resonance imaging (CMR) and assess their reproducibility in experienced and less experienced observers.

**Methods:**

Consecutive patients planned for CMR underwent RT3DE within 8 h of CMR with Philips (volumetric method) and Toshiba Artida (speckle tracking method). Left ventricular ejection fraction (LVEF), left ventricular end-diastolic volume (LVEDV) and end-systolic volume (LVESV) were measured using RT3DE, by four trained observers, and compared with CMR values.

**Results:**

Thirty-five patients were included (49.7 ± 15.7 years; 55 % male), 30 (85.7 %) volumetric and 27 (77.1 %) speckle tracking datasets could be analysed. CMR derived LVEDV, LVESV and LVEF were 198 ± 58 ml, 106 ± 53 ml and 49 ± 15 %, respectively. LVEF derived from speckle tracking was accurate and reproducible in all observers (all intra-class correlation coefficients (ICC) > 0.86). LVEF derived from the volumetric method correlated well to CMR in experienced observers (ICC 0.85 and 0.86) but only moderately in less experienced observers (ICC 0.58 and 0.77) and was less reproducible in these observers (ICC = 0.55). Volumes were significantly underestimated compared with CMR (*p* < 0.001).

**Conclusion:**

This study demonstrates that both RT3DE methodologies are sufficiently accurate and reproducible for use in daily practice. However, experience importantly influences the accuracy and reproducibility of the volumetric method, which should be considered when introducing this technique into clinical practice.

## Introduction

Left ventricular (LV) volumes and function are important parameters in clinical decision making [1–3]. Cardiac magnetic resonance imaging (CMR) is currently considered the reference standard [4–6]. However, echocardiography is the technique most frequently used in routine clinical practice, as it is cheaper, more readily available and more patient-friendly. Over the past decade real-time 3D echocardiography (RT3DE) has become integrated in clinical practice, also gaining applications in cardiac resynchronisation therapy and stress echocardiography [7–10]. Currently, different commercially available RT3DE methods are used, relying on different semi-automated contour detection algorithms. During the past decade, several studies have affirmed that volumetric method RT3DE is more accurate and reproducible than two-dimensional echocardiography for measurement of LV volumes and ejection fraction (LVEF) when compared with CMR [8, 11–14]. More recently, Kleijn et al. have shown similarly accurate and reproducible results for RT3DE using the speckle tracking method [15]. As usually seen when new techniques are introduced, virtually all studies were performed in RT3DE research centres, with often multiple years of experience and expert observers, which might overestimate the accuracy that can be achieved in routine clinical practice.

The current study aimed to assess the accuracy of two different, commercially available, RT3DE methodologies compared with CMR in routine clinical practice and assess their reproducibility in experienced and less experienced observers.

## Methods

Consecutive patients scheduled for CMR were considered for inclusion if >18 years and if willing and able to give informed consent. Patients with congenital heart defects or arrhythmia were excluded. Participants underwent RT3DE within 8 h of CMR with both Philips X5 (Philips Healthcare, Best, the Netherlands) and Toshiba Artida (Toshiba Medical Systems, Tokyo, Japan), which are routinely used in our department. The medical ethics review board approved the study protocol. Written informed consent was obtained from all patients.

## RT3D echocardiography

RT3DE examinations were performed with two different echocardiography systems, Philips-iE33 with ×5-1 5 MHz transducer (Philips Medical Systems, Best, the Netherlands) based on the direct 3D volumetric method, and Toshiba Artida-4D with PST-25SX 3 MHz transducer (Toshiba Medical Systems, Tokyo, Japan) based on the 3D speckle tracking method. For both methods, a full-volume scan was acquired from the apical four-chamber view focusing on the left ventricle and maximising volume rate. Respectively, 4R-wave (volumetric method) or 6R-wave (3D speckle tracking) triggered subvolumes were recorded during end expiratory breath holding.

## CMR

**Fig. 1:**
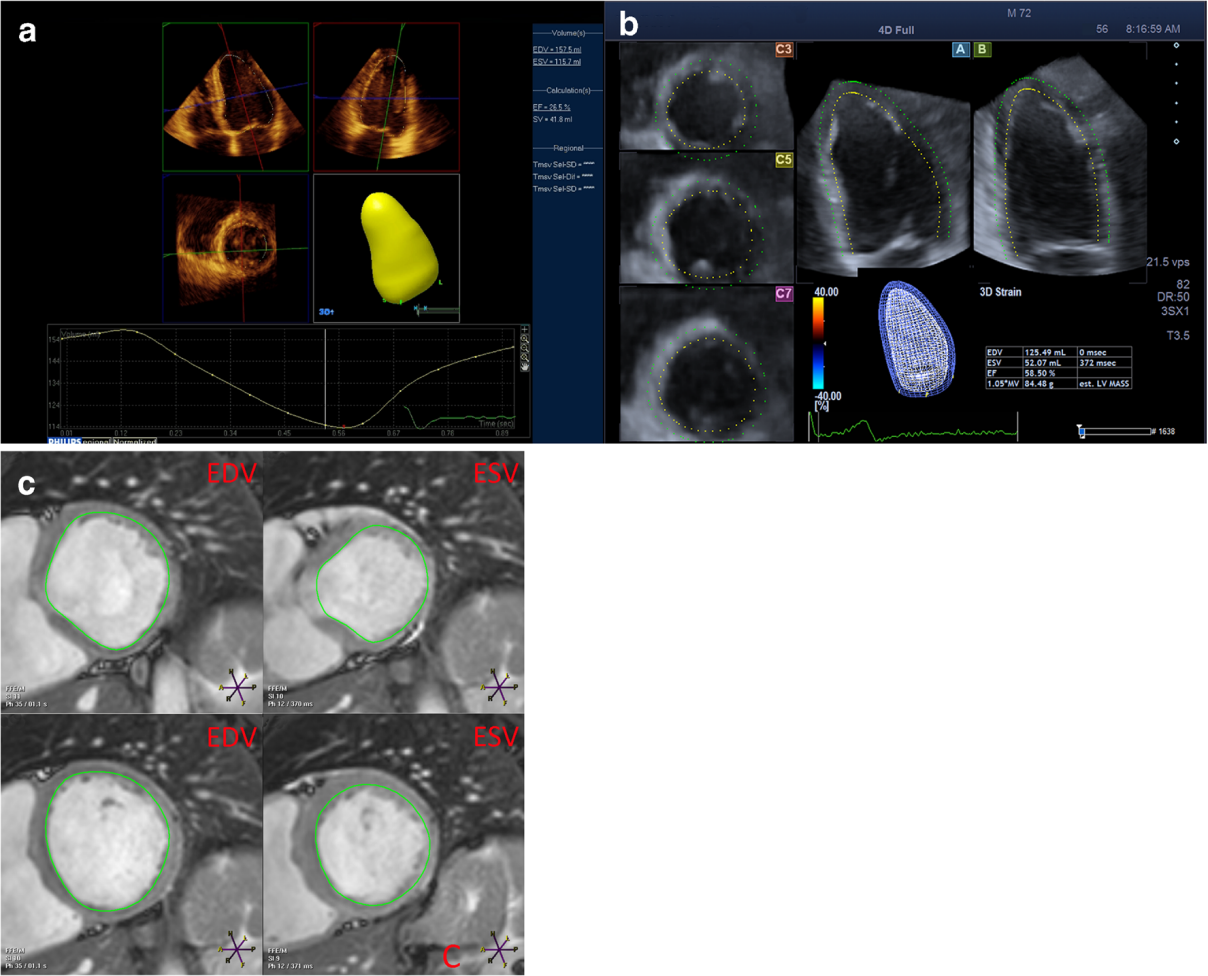
RT3DE and CMR workflow Workflow of both direct volumetric method (a) and speckle tracking method (b) RT3DE. In both methods contour detection is semi-automatic, requiring delineation of the mitral valve and apex in the end-diastolic view only (speckle tracking) or both end-diastolic and end-systolic views (volumetric). In Fig. 1C an example of contour tracing on multi-slice cine short axis of CMR is shown, the endocardial surface is traced in both end-diastole and end-systole

The CMR image acquisition and image analysis were performed in routine practice, by dedicated radiology technicians. Patients were imaged on a Philips Achieva 1.5-Tesla or Philips Ingenia 1.5-Tesla scanner (Philips Medical Systems, Best, the Netherlands). For all studies dedicated chest or torso phased array parallel-imaging capable surface coils were used with 12–28 elements. CMR images were acquired during repeated end-expiratory breath holds. Cine images were acquired using a retrospectively gated balanced steady state free precession (SSFP) sequence with 25–30 cardiac phases per cardiac cycle and a slice thickness of 8 mm without inter-slice gap. Sequences included LV four-chamber and LV two-chamber cine imaging, on which a multi-slice cine short axis was planned to include the entire left ventricle. Image analysis of LV end-diastolic volume (LVEDV), end-systolic volume (LVESV) and LVEF were performed manually using Philips Cardiac Explorer (Philips EWS (release 2.6), Philips Medical Systems, Best, the Netherlands). In all slices the endocardial borders were traced, basal slices were included in the volume if at least 50 % of the circumference of the LV cavity was surrounded by myocardial tissue (Fig. 1C). Papillary muscles were included in the blood volumes.

## RT3DE data analysis

### Volumetric method

Analysis was performed using dedicated vendor software, Qlab version 8.1 (Philips Medical Systems, Best, the Netherlands) for the volumetric method. The observer depicted the end-diastolic and end-systolic frame by visually identifying the image with the biggest and smallest LV cross-sectional area, respectively (Fig. 1A). Contour tracing was performed semi-automatically – using a geometry based border detection algorithm – by depicting the mitral valve annulus in the LV four-chamber and two-chamber views and the apex in either the four-chamber or two-chamber view in both the end-systolic and end-diastolic frame [16].

### Speckle tracking method

Analysis was performed using UltraExtend with Advanced Cardiac Package version 2.5 (Toshiba Medical Systems, Tokyo, Japan). The observer identified only the end-diastolic frame and for this frame contour tracing was performed by identifying the mitral annulus and apex in both four-chamber and two chamber views (Fig. 1B). Contour tracing is performed using imaging features – blocks of speckles – and is propagated from end-diastole to end-systole [16].

For both methods, datasets were excluded if the apex or mitral valve could not be depicted in the LV four- and two-chamber view. If necessary, manual adjustments to the semi-automatic contour tracing could be made in both software packages; however, this was minimised.

## Different observers

Four observers analysed all volumetric and 3D speckle tracking RT3DE datasets. All observers had received one-on-one training from experienced observers for half a day. It is important to note that the training and approach to analysis taught was similar for all observers [17]. Two observers were experienced in RT3DE (>3 months) and two observers were less experienced (1 month).

## Statistical analysis

A sample size of at least 28 was needed based on LVEF, using a bias to CMR of ≥5 % with a standard of 9 and β = 0.80 and α = 0.05. For continuous data means ± standard deviations (SD) or median and range were calculated as appropriate. Intra-class correlation coefficients (ICC) were calculated to assess the correlation of RT3DE and CMR, agreement was assessed with the Bland-Altman method. ICC assess the correlation within one class, for example the same measurement but with different methods. It therefore takes into account biases between measurements. ICC of >0.8 was considered excellent, 0.6–0.8 good, 0.5–0.6 moderate and <0.5 as poor correlation. Biases between RT3DE and CMR were expressed as mean ± SD and tested using a paired Student's *t* test with a two-tailed distribution or a Wilcoxon signed-rank test. Linear regression analysis was performed and a correlation plot was made for the observer with the best and the observer with the worst ICC between RT3DE and CMR. To obtain interobserver agreement, RT3DE results of all different observers were compared with each other using a paired Student’s *T*-test, ICC and Bland-Altman plot. A p value <0.05 was considered significant. Data analyses were performed using SPSS version 19.0 (SPSS, Inc., Chicago, Illinois).

## Results

Thirty-five patients were included in the study, mean age 49.7 ± 15.7 years, mean body surface area 1.92 ± 0.24 m^2^ and 19 (55 %) male. Indications for CMR were screening for cardiomyopathy in 11 (31 %), ischaemic heart disease in 6 (17 %), dilated cardiomyopathy in 4 (11 %), hypertrophic cardiomyopathy in 3 (9 %), other cardiomyopathies in 3 (9 %), pericarditis/myocarditis in 2 (6 %) and other in 6 (17 %) patients. For the volumetric method 30 of 35 (85.7 %) datasets were of sufficient quality to allow analysis, for the speckle tracking method 27 of 35 (77.1 %) datasets were of sufficient quality to be analysed. Average LVEDV, LVESV and LVEF were 198 ± 58 ml, 106 ± 53 ml and 49 ± 15 %, respectively, using CMR.

### Accuracy of RT3DE

**Fig. 2:**
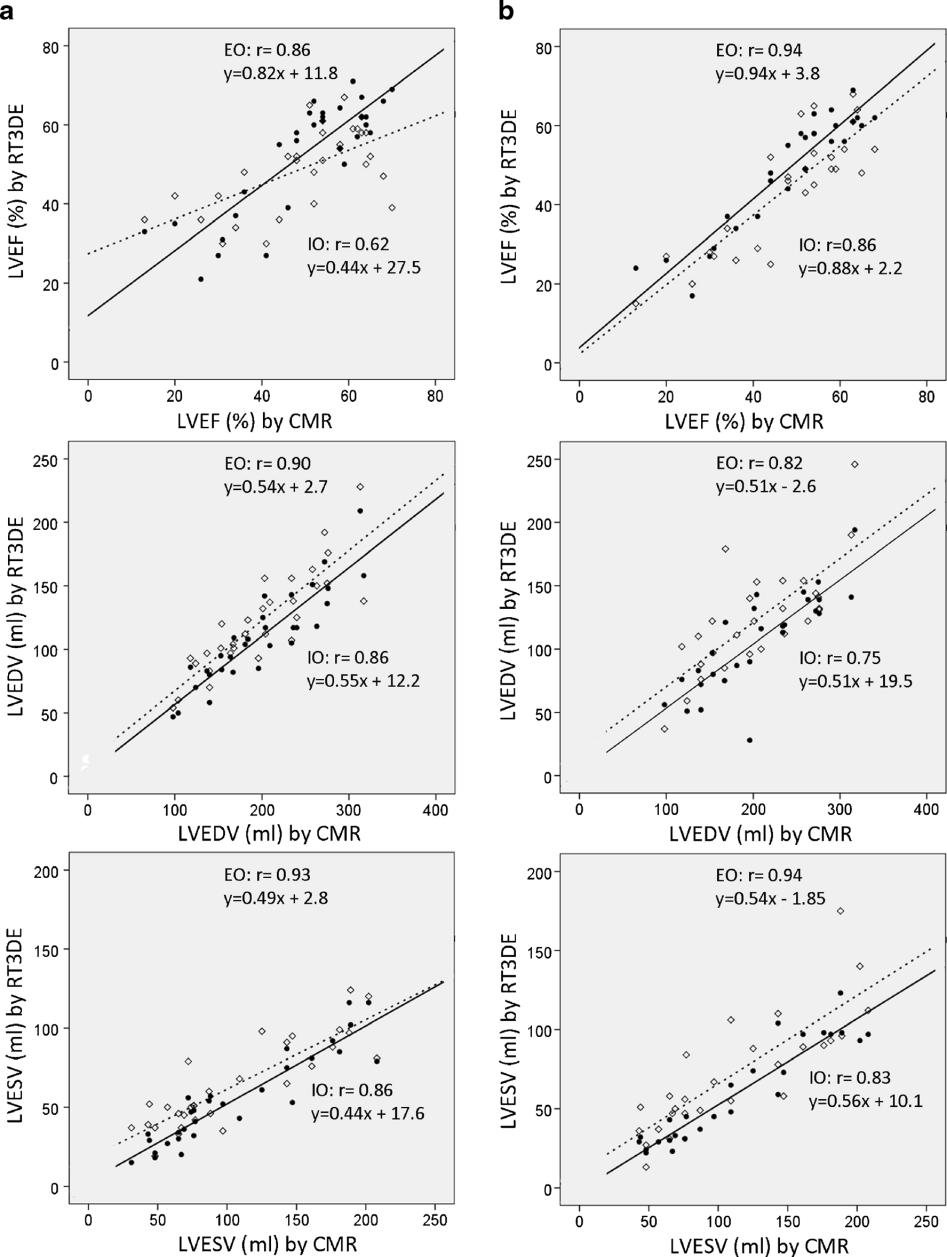
Correlation plots comparing direct volumetric method (**a**; DVM) and speckle tracking method (**b**; STM) with CMR measurements. From top to bottom depicting LVEF, LVEDV and LVESV. In each plot a correlation is shown for the best (experienced; EO=◆) and worst (inexperienced; IO=○) observer. Despite high correlations for LVEDV and LVESV, the regression formulas show that both methods increasingly underestimate CMR volumes as LV size grows. For DVM, RT3DE LVEF of the inexperienced observer was poorly associated to CMR

Correlations and agreement of all observers are listed in Table 1. LVEF derived from RT3DE speckle tracking method showed excellent correlations and reasonably good agreement with CMR in all observers. LVEF derived from the RT3DE volumetric method showed excellent correlation and reasonably good agreement to CMR in experienced, but only moderate correlation and agreement in inexperienced observers. In all observers, RT3DE derived LVEDV and LVESV were significantly underestimated compared with CMR by both methodologies. This is further illustrated by the correlation plots and linear regression analysis as shown in Fig. 2.

**Table 1:** Accuracy of RT3DE compared with CMR

	Observer 1		Observer 2		Observer 3		Observer 4	
	DVM	STM	DVM	STM	DVM	STM	DVM	STM
LVEDV								
Median [range]	107 [47–209]	116 [28–194]	112 [41–217]	96 [49–211]	116 [54–228]	100 [47–218]	110 [44–217]	122 [37–246]
ICC	0.79	0.73	0.75	0.75	0.78	0.79	0.79	0.70
Bias ± SD	−89 ± 32	−99 ± 38	−86 ± 36	−94 ± 37	−77 ± 34	−102 ± 34	−78 ± 34	−82 ± 41
*p*-value	<0.001	<0.001	<0.001	<0.001	<0.001	<0.001^#^	<0.001	<0.001
LVESV								
Median [range]	50 [15–116]	45 [22–123]	46 [13–113]	46 [19–167]	52 [19–124]	42 [19–158]	57 [11–137]	58 [13–175]
ICC	0.77	0.81	0.77	0.81	0.70	0.81	0.78	0.77
Bias ± SD	−51 ± 30	−53 ± 27	−49 ± 30	−49 ± 29	−42 ± 34)	−54 ± 28	−40 ± 30	−39 ± 32
*p*-value	<0.001^#^	<0.001^#^	<0.001^#^	<0.001^#^	<0.001^#^	<0.001^#^	<0.001^#^	<0.001^#^
LVEF								
Median [range]	58 [21–71]	56 [17–69]	55 [19–75]	52 [19–70]	51 [30–67]	53 [19–68]	48 [19–75]	48 [15–68]
ICC	0.85	0.94	0.86	0.89	0.58	0.90	0.77	0.86
Bias ± SD	2.9 ± 8.2	1.0 ± 5.2	2.5 ± 7.9	0.1 ± 7.0	−0.5 ± 11.7	0.5 ± 6.6	−2.1 ± 10.4	−3.6 ± 7.9
*p*-value	0.054^#^	0.306^#^	0.092	0.938	0.793	0.509^#^	0.280	0.259

The table lists the range of absolute left ventricular end-diastolic volume (LVEDV), end-systolic volume (LVESV) and left ventricular ejection fraction (LVEF) measured by direct volumetric method (DVM) and speckle tracking method (STM). The accuracy of both methods was compared with CMR in each observer using the intraclass correlation coefficient (ICC), the difference between RT3DE and CMR (bias) was calculated and tested for significance using a paired Student *T*-test or Wilcoxon signed-rank test as indicated by #

### Interobserver agreement

**Fig. 3:**
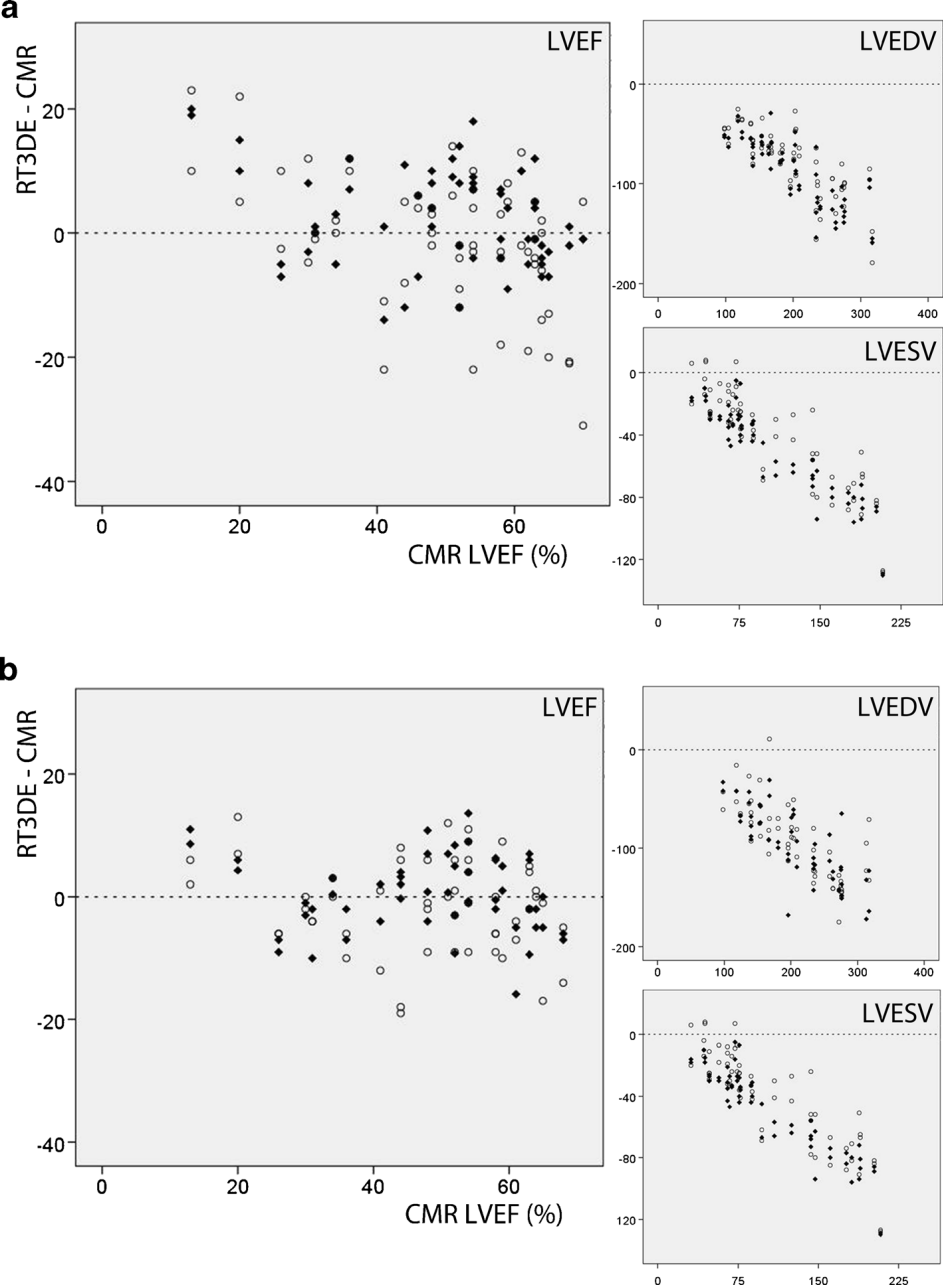
Bland-Altman plots including the measurements by all four observers in *one* plot. This figure visually illustrates the dispersion between all observers for *one* CMR value. On the y-axis the absolute difference between RT3DE and CMR measurements is depicted and on the x-axis the CMR derived value. As these are not measurements of one single observer but of multiple observers, limits of agreement and bias cannot be indicated in this figure 1a: volumetric method, 1b: speckle tracking method. ○ = inexperienced observers and ◆ = experienced observers

The interobserver agreement for LVEDV, LVESV and LVEF of experienced observers (observers 1 + 2) was good to excellent for both methods and is shown in Table 2. The interobserver agreement for the less experienced observers (observer 3 + 4) differed between the two methodologies. The speckle tracking method showed excellent correlation and good agreement but with a significant bias in the inexperienced observers for all LV parameters. For the volumetric method there was only a moderate correlation for LVEF, with a large but non-significant bias. Fig. 3 illustrates the differences between RT3DE and CMR derived measurements for both the experienced (observer 1 + 2) and less experienced (observer 3 + 4) observers. For LVEF the variation between observers is larger for the volumetric method (Fig. 3, top) than for the speckle tracking method (Fig. 3, bottom). For both methods the difference between RT3DE and CMR derived LVEDV and LVESV is more pronounced in patients with larger LVEDV.

**Table 2:** Interobserver variability of experienced vs inexperienced observers

	Experienced interobserver		Inexperienced interobserver	
	ICC	Bias ± SD	ICC	Bias ± SD
Direct volumetric				
LV EDV (ml)	0.93	3.3 ± 14.4	0.92	1.5 ± 16.8
LV ESV (ml)	0.90	1.7 ± 13.2	0.84	−1.9 ± 17.5
LV EF (%)	0.78	−0.4 ± 9.8	0.55	1.5 ± 12.6
Speckle tracking				
LV EDV (ml)	0.82	4.4 ± 24.0	0.80	20.7 ± 25.7*
LV ESV (ml)	0.85	3.5 ± 19.1	0.85	15.3 ± 19.4*
LV EF (%)	0.94	−1.0 ± 5.2	0.87	−4.1 ± 6.9*

intraclass correlation coefficients and mean difference ± standard deviation; *p < 0.05 using paired Student *T*-test

## Discussion

The current study was the first to directly compare the volumetric and speckle tracking method to CMR in the same group of patients and demonstrated that both methodologies are sufficiently accurate and reproducible to use in daily practice. However, the speckle tracking method was slightly more accurate and reproducible, most markedly in less experienced observers. As previously demonstrated, LV volumes were underestimated with both RT3DE methods compared with CMR and absolute values should not be relied on.

### Accuracy of RT3DE in daily practice

The accuracy of RT3DE derived LVEF in our study was less favourable than reported in most previous studies comparing RT3DE with CMR that were conducted in a research setting [11–14]. In our study, the mean difference ± SD compared with CMR was 2.9 ± 8.2 % for the volumetric and 1.0 ± 5.2 % for the speckle tracking method, compared with differences of 0  ±  3 % for the volumetric method in the studies by Soliman et al. and Jenkins et al., which were performed in centres with high RT3DE expertise levels [11, 12]. This is not different from CMR measurement of LVEF, where difference in the intra-observer bias of highly experienced and less-experienced observers, reported by Caudron et al., is within a similar range [18]. Furthermore, some of the previous reports only included patients with good to excellent image quality. Our study focussed on the daily clinical practice, in which many patients have suboptimal image quality; datasets were only excluded if one of the anatomic landmarks could not be defined.

### Volumetric vs speckle tracking method

The current report is the first to directly compare both the volumetric and speckle tracking method to CMR. We found that the speckle tracking method was more accurate and, in contrast to the study by Kleijn et al., more reproducible for RT3DE LVEF than the volumetric method (Table 1+2), most notably in the less experienced observers [19]. Both methodologies use semi-automatic contour detection, however based on entirely different software principles. The volumetric method uses a geometry based border detection algorithm, using surface detection based on modelled assumptions in a coarse-to-fine order. The speckle tracking method does not use border detection in subsequent frames but propagates contours from frame to frame predominantly using imaging features – blocks of speckles – to do this [16]. In our experience, the volumetric method RT3DE automated contour detection did not perform optimally in end-systolic frames, even when image quality was good, requiring a higher degree of manual corrections compared with the speckle tracking method. These manual corrections will likely have influenced the reproducibility. It is important to note that more datasets could be analysed for the volumetric (86 %) than for the speckle tracking method (77 %), which is an important drawback especially in dilated left ventricles.

### LV volume underestimation

Both RT3DE methods underestimate LVEDV and LVESV, with underestimation being slightly more pronounced for the speckle tracking method. Previous studies have also reported important underestimation using RT3DE, but the degree of underestimation varies widely, for LVEDV from −7  ±  20) ml to −67  ±  46) ml [11, 20]. These differences might not only be explained by differences in the RT3DE techniques used, but partly also be due to differences in CMR analysis. For example, inclusion or exclusion of papillary muscles is rarely specified in reports but importantly influences LV volumes [21]. The biases in LV volumes reported in more recently published studies most closely resemble our data [20, 22, 23]. These studies, like ours, used an unselected patient population. Like Moceri et al., we found that underestimation of RT3DE augments with increasing LVEDVs (Fig. 2 & 3) [23]. Since dilated ventricles are often the ventricles that we are interested in, this is a serious drawback for the use of RT3DE. However LVEF – which can be measured reliably with RT3DE – is the most important parameter determining therapeutic strategy and adverse outcomes such as rehospitalisation and death [1–3]. We advocate the use of RT3DE for assessment of LV function not only in research settings, but also in clinical practice.

#### Limitations

Only 30 % of the participants included in the analysis had an LVEF <45 %, which was partly because the image quality in severely dilated left ventricles was poor. Even though datasets were only excluded if the anatomic landmarks – required for contour detection – could not be defined at all, still 14 % of the volumetric datasets and 23 % of the speckle tracking datasets were excluded. This is in accordance with previous studies which had on average 15 % poor image quality.[8] However, this is the group of patients with already dilated left ventricles in which monitoring of LVEF is most important. Further improvements by vendors, in transducer capacity and software, aimed at increasing the imaging angle, overall and lateral resolution are still mandatory to reduce this problem.

## Conclusion

RT3DE, using both the volumetric and the speckle tracking method, can be used to reliably assess LVEF in daily practice for clinical decision-making (i.e. prognosis, indication for ICD). However, image quality is insufficient for analysis in part of the patient group and experience importantly influences the accuracy and reproducibility of the volumetric method, both of which should be considered before introducing this method into routine daily practice.
